# Consumptive and nonconsumptive effect ratios depend on interaction between plant quality and hunting behavior of omnivorous predators

**DOI:** 10.1002/ece3.2828

**Published:** 2017-03-09

**Authors:** Jörg G. Stephan, Johan A. Stenberg, Christer Björkman

**Affiliations:** ^1^ Department of EcologySwedish University of Agricultural SciencesUppsalaSweden; ^2^ Department of Plant Protection BiologySwedish University of Agricultural SciencesAlnarpSweden

**Keywords:** antipredator behavior, biological control, clutch size frequency distribution, foraging behavior, host acceptance, indirect plant defense, nonlethal predator effects, plant suitability, predator–prey interactions, trait‐mediated effects

## Abstract

Predators not only consume prey but exert nonconsumptive effects in form of scaring, consequently disturbing feeding or reproduction. However, how alternative food sources and hunting mode interactively affect consumptive and nonconsumptive effects with implications for prey fitness have not been addressed, impending functional understanding of such tritrophic interactions. With a herbivorous beetle, two omnivorous predatory bugs (plant sap as alternative food, contrasting hunting modes), and four willow genotypes (contrasting suitability for beetle/omnivore), we investigated direct and indirect effects of plant quality on the beetles key reproductive traits (oviposition rate, clutch size). Using combinations of either or both omnivores on different plant genotypes, we calculated the contribution of consumptive (eggs predated) and nonconsumptive (fewer eggs laid) effect on beetle fitness, including a prey density‐independent measure (c:nc ratio). We found that larger clutches increase egg survival in presence of the omnivore not immediately consuming all eggs. However, rather than lowering mean, the beetles generally responded with a frequency shift toward smaller clutches. However, female beetles decreased mean and changed clutch size frequency with decreasing plant quality, therefore reducing intraspecific exploitative competition among larvae. More importantly, variation in host plant quality (to omnivore) led to nonconsumptive effects between one‐third and twice as strong as the consumptive effects. Increased egg consumption on plants less suitable to the omnivore may therefore be accompanied by less searching and disturbing the beetle, representing a “cost” to the indirect plant defense in the form of a lower nonconsumptive effect. Many predators are omnivores and altering c:nc ratios (with egg retention as the most direct link to prey fitness) via plant quality and hunting behavior should be fundamental to advance ecological theory and applications. Furthermore, exploring modulation of fitness traits by bottom‐up and top‐down effects will help to explain how and why species aggregate.

## 1 Introduction

Top‐down effects of predators on prey consist of two components: a direct consumptive and an indirect nonconsumptive effect associated with changes in prey behavior. Nonconsumptive effects can have far‐reaching impacts on trophic cascades (Beckerman, Uriarte, & Schmitz, 1997; Trussell, Ewanchuk, & Bertness, 2003), ecosystem functions (Matassa & Trussell, 2011; Schmitz, Grabowski, & Peckarsky, 2008), and often equal or exceed the effects of consumption (Preisser, Bolnick, & Benard, 2005; Schmitz, Krivan, & Ovadia, 2004). Nonconsumptive effects can increase prey vulnerability to other mortality factors (McCauley & Rowe, 2011) or generate physiological stress, resulting in energetic costs with a cascading negative impact on prey reproduction (Creel, Winnie, & Christianson, 2009; Nelson, 2007; Nelson, Matthews, & Rosenheim, 2004). Lower reproduction due to predators caused by, for example, mating interruption (Travers & Sih, 1991), higher conspicuousness of males attracting females (Uzendoski, Maksymovitch, & Verrell, 1993), or changes in prey behavior that result in lower weight gain or poorer provisioning of progeny (Harfenist & Ydenberg, 1995) should represent the strongest nonconsumptive effects as they reduce prey fitness.

Here, we investigate key reproductive traits (clutch size, oviposition rate) of a herbivorous leaf beetle and examine whether these traits are altered by bottom‐up (host plants of different quality) and top‐down (individual and combined effect of predators with different hunting behavior) effects.

Bottom‐up effects on herbivores depend on plant quality, including plant resistance (Karban, 2011; Schaller, 2008). Variation in plant quality exists among species, but also between plant genotypes (Kaplan & Thaler, 2010; Stenberg, Lehrman, & Björkman, 2011a), and affects herbivore performance (Kaplan & Thaler, 2010), fitness (Lehrman, Torp, Stenberg, Julkunen‐Tiitto, & Björkman, 2012) and ultimately community composition (Schmitz et al., 2008; Wimp, Murphy, Finke, Huberty, & Denno, 2010) via differences in, for example, trichomes that hinder foraging/moving (Mulatu, Applebaum, & Coll, 2006) or volatiles functioning as infochemicals (Degen, Dillmann, Marion‐Poll, & Turlings, 2004).

The impact of plant genotype on higher trophic levels has also been addressed (Abdala‐Roberts et al., 2016; Kabir, Moritz, & Stenberg, 2014; Tack, Ovaskainen, Pulkkinen, & Roslin, 2010), and attempts have been made to link nonconsumptive effects of predators to the plant genotype upon which the interaction occurs (Kersch‐Becker & Thaler, 2015; Thaler, Contreras, & Davidowitz, 2014). Strongest effect, however, should occur if the predator is an omnivore and differing plant food quality associated with the genotype can alter the means to satisfy nutritional needs, leading to higher or lower consumption of herbivorous prey (Lundgren, Hesler, Tilmon, Dashiell, & Scott, 2009; Stenberg, Lehrman, & Björkman, 2011b).

Herbivore traits altered by the nonconsumptive effect of a single predator type that have been investigated mostly include performance parameters (feeding, growth, body mass). However, per capita population growth and number of offspring in a population have been looked at Thaler et al. (2014) and Kersch‐Becker and Thaler (2015). However, no attempts have been made to tease apart the contributions of consumptive and nonconsumptive effects on fitness of individual prey and to gain a mechanistic understanding of how the nonconsumptive effect is changed by trophic levels below the prey. This plant‐driven change of the omnivore and the prey behavior may provide general insights into such tritrophic interactions, possibly also important for true predators.

The first trait of insect herbivores that we investigate is clutch size, which may depend on bottom‐up (Kagata & Ohgushi, 2002; Pilson & Rausher, 1988), lateral (Stephan, Stenberg, & Björkman, 2015), or top‐down factors (Siemens & Johnson, 1992; Subinprasert & Svensson, 1988). Assuming that females are not exclusively constrained to maximize realized fecundity (Clark & Faeth, 1998), they should place more eggs in the same clutch/on the same plant to increase the ability to overcome different plant defenses (Young & Moffett, 1979) or lower the encounter probability of predators as increased time spent searching for prey leads to lower consumption (Vine, 1971). Such general mechanistic explanations serve as the basis for, for example, the group defense hypothesis in insects (Hunter, 2000).

Besides prey behavior, predator foraging behavior determines whether larger clutch sizes are advantageous. Different hunting modes (Miller, Ament, & Schmitz, 2014) employed by predators can lead to an increased chance of survival in larger clutches if, for example, the predator does not consume all encountered eggs immediately (Stephan, Low, Stenberg, & Björkman, 2016). On the other hand, placing too many eggs at one location will increase exploitative competition between the hatching larvae (Mitchell, 1975) and force them to migrate, in turn increasing predation risk (Matsumoto, 1990). In addition to changing the mean, the frequency distribution of clutch sizes may change due to resource abundance (Kagata & Ohgushi, 2002) or predator protection (Atsatt, 1981). Frequency changes in response to the host plant quality or predator presence should affect egg survival. How insect herbivore clutch size, but especially clutch size frequency distribution, is interactively affected by predator foraging behavior and plant quality have not been addressed previously.

The second trait we investigate is oviposition rate, that is, if it is correlated with other reproductive traits (Lehrman et al., 2012), a proxy for fitness that can change, for example, because of temperature (Tammaru, Kaitaniemi, & Ruohomäki, 1996), habitat plant species richness (Unsicker et al., 2010), or intraspecific exploitative competition (Hemptinne, Dixon, & Coffin, 1992). As a fitness parameter, it is possible to calculate the number of eggs not laid due to plant genotype (host acceptance) or omnivore presence (presence vs. absence). By relating the number of eggs not laid due to omnivore presence to the number of predated eggs, it is possible to calculate a consumptive: nonconsumptive effect ratio (c:nc ratio) that is independent of prey density (that will vary due to the plant genotype). Here, we use this ratio to investigate how host plant quality (with contrasting effects on herbivore and omnivore) and omnivore type/combination interactively shape the contributions of consumptive and nonconsumptive effects on prey fitness.

The well‐studied system examined herein provides key components that make it particularly suitable for studying effects of plant genotypes, nonconsumptive effects, and their interaction. Genotypes of naturally hybridizing willows deliver different levels of suitability to the specialist leaf beetle *Phratora vulgatissima,* and we use four bred willow genotypes that are among the most suitable and unsuitable for the leaf beetle (Stenberg, Lehrman, & Björkman, 2010). This beetle is able to reduce its oviposition rate according to how many conspecifics have visited the plant individual, while the number of conspecific eggs seems to be of less importance (Stephan et al., 2015). At the same time, the relative consumption of plant (sap) and animal food (leaf beetle eggs) by two of the most important omnivores (*Anthocoris nemorum* and *Orthotylus marginalis*) changes in parallel with plant sap quality of genotypes (Liman, Dalin, & Björkman, 2016). These omnivores are well studied, and we (1) can exclude plant defenses like trichomes to affect the omnivores (Björkman & Ahrne, 2005), be fairly certain that omnivores are less motivated to forage for clutches if they can more easily consume plant sap (Stenberg et al., 2011a; Stephan, Low, et al., 2016), (2) can assume that plant architecture is less important than plant sap quality to alter the behavior (Stephan, Low, et al., 2016), and (3) showed that the two omnivores show distinctly different foraging modes (Björkman, Dalin, & Eklund, 2003) and interspecific interactions does not affect the total predation rate (Björkman & Liman, 2005). One of the omnivores does not consume all eggs discovered in a clutch (“run‐and‐eat”), which results in changed egg survival probabilities in differently sized egg clutches. In contrast, the other omnivore exhibits “find‐and‐stay” behavior and egg survival is independent of clutch size. Therefore, the aggregation behavior of the leaf beetle interacts with the omnivore hunting mode, and this interaction also depends on the host plant quality as the strength of the underlying mechanism, attack abatement, changes with quality of alternative food available to the omnivore (Stephan, Low, et al., 2016). However, whether and how the leaf beetles change their aggregation behavior due to omnivore presence is unknown.

We hypothesize that the leaf beetle females lay larger egg clutches and increase the proportion of larger clutches on unsuitable plant genotypes (1) and in the presence of the “run‐and‐eat” omnivore (2) as larger clutches would increase egg survival. We also hypothesize that omnivore presence will induce a lower oviposition rate (3) as the female beetles try to minimize egg losses to predation. The lower oviposition rate will depend on omnivore species, and plant genotype, ultimately changing the contributions of consumptive and nonconsumptive effects to beetle fitness (changed c:nc ratio) (4).

## 2 Material and Methods

### 2.1 Study system

The adults and larvae of the herbivore *Phratora vulgatissima* L. (Coleoptera: Chrysomelidae) skeletonize the leaves of their natural host plant, willows (*Salix* spp.). It is the most important specialist herbivore of willow in Europe (Peacock & Herrick, 2000; Peacock, Hunter, Turner, & Brain, 2002), frequently reaching outbreak densities in natural willow stands and plantations (Björkman, Bengtsson, & Häggström, 2000; Dalin, Kindvall, & Björkman, 2009). In plantations, used for biomass production, outbreaks can reduce growth by up to 40% (Björkman, Höglund, Eklund, & Larsson, 2000). Adults overwinter in shelter‐providing vertical objects like reeds or trees with aging bark (Björkman & Eklund, 2006), emerge in April, feed for about 2 weeks, mate and subsequently lay hundreds of eggs on the underside of leaves in clutches of 1–50. Larvae hatch after 15–20 days and feed gregariously on leaves during the first and second instars and then solitarily during the third instar (Kendall, Wiltshire, & Butcher, 1996). Larvae pupate in the soil; adults emerge in August and after a short period of feeding and find hibernation sites.

*Anthocoris nemorum* L. (Heteroptera: Anthocoridae) is an important predator of *P. vulgatissima* because it can consume large numbers of beetle eggs (Björkman et al., 2003) and is an effective biological control agent in apple orchards (Sigsgaard, 2010). Like most other predatory heteropteran bugs, *A. nemorum* also feeds on shallowly located fluids from the green parts of host plants. However, it is mainly regarded as a predator (Lauenstein, 1979).

*Orthotylus marginalis* Reut. (Heteroptera: Miridae) also consumes large numbers of *P. vulgatissima* eggs (Björkman et al., 2003) and has been observed to be mainly predacious (Lehman, 1932), while other observations support the impression that it can survive on a minimal amount of animal food but that it has a preference for this source of nutrition (Kullenberg, 1944).

Beetles (collected from willow trees) and omnivores (caught with a swipe net on ground vegetation) came from natural populations in the Uppsala region of Sweden. The communal living gregarious beetles exhibit a synchronized development and the individuals used here were therefore even aged and within their first weeks of egg laying, with stable oviposition rates for several weeks (Lehrman et al., 2012). While the omnivores were starved over night before release in the cages, the beetles were provided with shoots from a preferred natural willow, *S. cinerea*. The *Salix* genotypes (78021 (*S. viminalis*), 78183 (*S. viminalis*), Gudrun (*S. burjatica* × *S. dasyclados*), Loden (*S. dasyclados*)) selected for the experiments were chosen because they differ in chemical composition, thus affecting their suitability for both the leaf beetle and the omnivores. The suitability for the leaf beetle (fecundity) has been found to increase in the order Gudrun < Loden < 78021 < 78183 (Stenberg et al., 2010), whereas the suitability for *A. nemorum* in the absence of prey follows the reverse order. When prey is present, the most suitable of these genotypes for *A. nemorum* (fecundity) is genotype 78183, while the suitability of the genotypes Gudrun, Loden, and 78021 is similar (Stenberg et al., 2011a). Although not so detailed information is available for *O. marginalis,* we showed that plant quality is affecting this omnivore in similar ways (Stephan, 2016; Stephan, Low, et al., 2016).

Clones of the different genotypes were grown in the glasshouse where the experiments were subsequently performed (23°C, RH 80, L18:D6). Plants were of similar age, had between 17 and 35 leaves, and were randomly assigned to the treatments. At least 1 day (to exclude green leaf volatiles and fresh wounds) before each experiment, plants were prepared by removing the top 2–4 newly emerged, incompletely unfolded leaves and the lowest old and withering leaves to standardize the shoots. All plants had approximately the same cumulative leaf area because smaller leaves are compensated for by a higher number of leaves on a plant (Stephan et al., 2015).

### 2.2 Oviposition in the presence and absence of different omnivores

Two (to reduce beetle‐specific variability, although hereafter we will still refer to effects on individual fitness) ovipositing *P. vulgatissima* were allowed to lay eggs for 6 days on the prepared plants in cylindrical transparent plastic cages (70 cm height, 30 cm diameter) with a net on top. Six days is the maximum duration egg predation can be measured as the eggs start to hatch under the experimental conditions after seven to 8 days. This treatment with only the leaf beetles served as control, and, as in all treatments, we set up at least seven plant individuals (replicates). However, host acceptance was very low (we only used plants that received eggs) for the very unsuitable genotype Gudrun leading to fewer replicates (78183/78021/Loden: *N* = 10, Gudrun: *N* = 6). Five treatments were used by additionally caging the following omnivore individuals with the two leaf beetles: two *A. nemorum* (2 AN; 78183: 10, 78021: 8, Loden: 8, Gudrun: 4); two *O. marginalis* (2 OM; 78183: 8, Loden: 7); one *A. nemorum* plus one *O. marginalis* (1 AN 1 OM; 78183: 8, Loden: 7); and four *O. marginalis* (4 OM; 78183: 7, Loden: 8). Although only with one omnivore, the last treatment was included to give an idea of how predator density might affect the extent of the nonconsumptive effect. The experiments involving the Control and the 2 AN treatment were performed in 2009 (hereafter, part one), while the remaining treatments were performed in 2015 (hereafter, part two) under the same conditions (including glasshouse and collection of species). The experiment was performed at a time of the year when *A. nemorum* adults and nymphs of *O. marginalis* can be found. Due to the late development of *A. nemorum* adults in 2015 (resulting in a slightly later experimental date), many nymphs of *O. marginalis* had already reached their third or fourth instar, instead of most being first and second instar, as in 2009. Both omnivores feed by sucking and leave empty egg shells behind. Thus, we were able to count shells and calculate the proportion of eggs surviving per clutch and the number of eggs initially laid in a clutch/on a plant.

### 2.3 Calculation of nonconsumptive effect on reproductive output

We used the proportion of consumed eggs (number of eggs predated with omnivore present divided by number of eggs laid with omnivore present) to calculate the consumptive effect and the proportion of eggs not laid due to omnivore presence (number of eggs laid with omnivore present divided by number of eggs laid without omnivore) to calculate the nonconsumptive effect. To relate the contributions of consumptive and nonconsumptive effect to the overall omnivore effect on the prey, independent of prey density, we also calculated the consumptive: nonconsumptive ratio (c:nc ratio). In contrast to the statistical analysis, these comparisons were made using the control from part one of the experiment in relation to the omnivore treatments in part two, which we think is valid as ratios of ratios are compared and we are careful when making quantitative statements.

### 2.4 Data analysis

The count and survival data were analyzed with generalized linear mixed models with the plant individual as a random effect. We also included a random effect for every observation nested within the plant. For the proportion of survival within clutches and within all eggs laid on a plant (predated/survived), we used a binomial distribution with logit link, and for the count data (clutch size, eggs on plant), we used a Poisson distribution with a log link function. Because the two parts of the experiment were performed in different years (and the genotype–treatment combinations are not complete), we did not pool these data and modeled the experimental parts separately. In addition to the effect of the plant genotype/omnivore treatments on the mean clutch size, we were also interested in the effect on the clutch size distribution. Therefore, we included the number of eggs laid on a plant in the models investigating the clutch size and compared the slopes (by altering the reference level of the model). Because data were limited for genotypes with low host acceptance, we did not start with a model including the interaction between eggs laid, treatment, and plant genotype but rather with only two‐way interactions followed by step wise removing of nonsignificant interactions/main effects. Besides investigating the change in the mean clutch size with models that assign Poisson distributions to every count, we were also interested in the actual distribution of clutch sizes. We therefore performed Kolmogorov–Smirnov tests (KS test) and illustrated the point of maximum separation (D‐values) between the two distributions. Because these comparisons use the relative distribution of the data and are therefore independent of the number of eggs laid (which may vary between the years and therefore between the two parts of the experiment), we related the distributions of all omnivore treatments to the control.

## 3 Results

**Figure 1:**
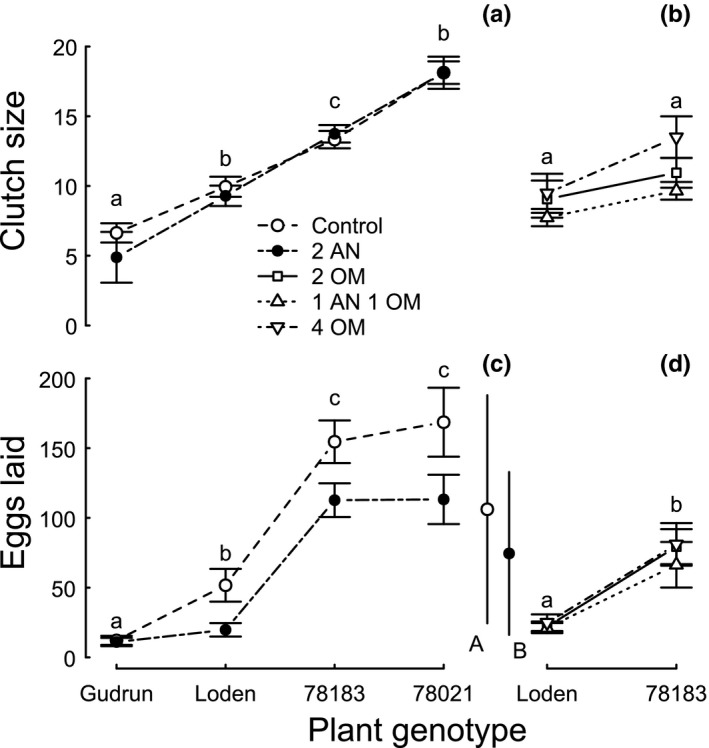
Mean (±SE) clutch size and eggs laid on individual plants by two *Phratora vulgatissima* females on four different *Salix* genotypes (*S. dasyclados*: Gudrun, Loden; *S. viminalis*: 78183, 78021) and the presence of omnivores (Control = only leaf beetles, AN = *Anthocoris nemorum*, OM = *Orthotylus marginalis*) for the first (a, c) and second (b, d) parts of the experiment. Lowercase letters indicate differences between genotypes and uppercase letters differences between overall means (±SD) of treatments (*p* < .05; Tukey's test)

The omnivore treatments had no effect, but plant genotype strongly modulated the mean clutch size of *P. vulgatissima* (Table 1: M1, M2) with the *S. viminalis* genotype 78021 associated with the largest clutches (Figure 1a) in the presence of *A. nemorum* in the first part of the experiment. In the second part of the experiment, the mean clutch size did not differ between treatments, but seemed to resemble the genotype‐specific sizes recorded during the first part. In general, clutches were larger if more eggs were laid on a plant and this relationship became increasingly stronger in the presence of *A. nemorum* in the first part (Figure S1) and tended to be stronger on the Loden genotype compared to 78183 in the remaining omnivore treatments (Figure S2).

**Table 1:** Analysis‐of‐deviance tables (type III test) from generalized linear mixed models investigating the oviposition behavior of the leaf beetle *Phratora vulgatissima* and how it is altered by the presence of different omnivores (AN = *Anthocoris nemorum*, OM = *Orthotylus marginalis*) and the plant genotype on which the interaction occurred (*S. dasyclados*: Gudrun, Loden; *S. viminalis*: 78021, 78183). Genotype = plant genotype; Treatment = always two leaf beetles with varying combination and number of omnivores; Eggs laid = cumulative number of eggs on a plant. Nonsignificant terms (italicized) were removed stepwise from the final model starting from the bottom row. “/” means “nested within”; Obs = each observation

Model	Model type	Random factor	Response variable	Explanatory variables	χ^2^	*df*	AIC	$R_{\mathrm{GLMM}(m)}^{2}$	$R_{\mathrm{GLMM}(c)}^{2}$	*p*‐Value
Does the mean clutch size depend on the omnivore treatment (Control, 2 AN) or the plant genotype (Gudrun, Loden, 78021, 78183)?										
M1	GLMM (Poisson)	Plant/Obs	Clutch size	Intercept	160.83	1	2799.12	.28	.33	<.001
				Genotype	42.69	3	2799.12	.28	.33	<.001
				Treatment	2.66	1	2799.12	.28	.33	.10
				Eggs laid	1.20	1	2799.12	.28	.33	.27
				Treatment × Eggs laid	5.07	1	2799.12	.28	.33	.02
				Genotype × Eggs laid	*4.83*	*3*	*2800.29*	*.29*	*.33*	*.18*
				Genotype × Treatment	*4.20*	*3*	*2802.17*	*.30*	*.34*	*.23*
Does the mean clutch size depend on the omnivore treatment (2 OM/1 AN 1 OM/4 OM) or the plant genotype (Loden, 78183)?										
M2	GLMM (Poisson)	Plant/Obs	Clutch size	Intercept	286.40	1	1347.03	.11	.12	<.001
				Genotype	0.60	1	1347.03	.11	.12	.43
				Treatment	3.49	2	1347.03	.11	.12	.17
				Eggs laid	14.94	1	1347.03	.11	.12	<.001
				Genotype × Eggs laid	*3.36*	*1*	*1345.74*	*.12*	*.13*	*.06*
				Treatment × Eggs laid	*3.59*	*1*	*1345.77*	*.14*	*.14*	*.16*
				Genotype × Treatment	*0.86*	*2*	*1348.88*	*.14*	*.14*	*.64*
Does the eggs laid per plant depend on the omnivore treatment (Control, 2 AN) or the plant genotype (Gudrun, Loden, 78021, 78183)?										
M3	GLMM (Poisson)	Plant	Eggs laid	Intercept	955.53	1	657.50	.77	.77	<.001
				Genotype	190.43	3	657.50	.77	.77	<.001
				Treatment	11.35	1	657.50	.77	.77	<.001
				Genotype × Treatment	*4.24*	*3*	*659.38*	*.78*	*.78*	*.23*
Does the eggs laid per plant depend on the omnivore treatment (2 OM/1 AN 1 OM/4 OM) or the plant genotype (Loden, 78183)?										
M4	GLMM (Poisson)	Plant	Eggs laid	Intercept	557.70	1	408.42	.51	.51	<.001
				Genotype	45.72	1	408.42	.51	.51	<.001
				Treatment	*1.21*	*2*	*411.22*	*.52*	*.52*	*.54*
				Genotype × Treatment	*0.46*	*2*	*414.75*	*.53*	*.53*	*.79*
Does the survival (2 AN) within clutches depend on the clutch size and the plant genotype (Gudrun, Loden, 78021, 78183)?										
M5	GLMM (Binomial)	Plant/Obs	Survival (survived/predated)	Intercept	0.05	1	522.41	.25	.29	.81
				Clutch size	1.18	1	522.41	.25	.29	.27
				Genotype	2.33	3	522.41	.25	.29	.50
				Clutch size × Genotype	8.89	3	522.41	.25	.29	.03
Does the survival (2 OM/1 AN 1 OM/4 OM) within clutches depend on the treatment, the clutch size and plant genotype (Loden, 78183)?										
M6	GLMM (Binomial)	Plant/Obs	Survival (survived/predated)	Intercept	*13.40*	*1*	*317.29*	*.08*	*.11*	<.001
				Clutch size	*0.02*	*1*	*317.29*	*.08*	*.11*	*.86*
				Genotype	*3.73*	*1*	*317.29*	*.08*	*.11*	*.05*
				Treatment	*3.35*	*2*	*317.29*	*.08*	*.11*	*.18*
				Clutch size × Treatment	*11.59*	*2*	*317.29*	*.08*	*.11*	<.01
				Genotype × Treatment	*4.06*	*2*	*343.68*	*.37*	*.40*	*.13*
				Clutch size × Genotype	*0.08*	*1*	*332.96*	*.38*	*.38*	*.76*
				Clutch size × Genotype × Treatment	*2.90*	*2*	*346.52*	*.61*	*.62*	*.23*
Does the survival (2 AN) of eggs on a plant depend on the number of eggs on the plant and the plant genotype (Gudrun, Loden, 78021, 78183)?										
M7	GLMM (Binomial)	Plant/Obs	Survival (survived/predated)	Intercept	*6.64*	*1*	*198.73*	*.25*	*.25*	<.01
				Genotype	*18.58*	*3*	*198.73*	*.25*	*.25*	<.001
				Eggs on plant	*2.46*	*1*	*198.50*	*.28*	*.28*	*.11*
				Eggs on plant × Genotype	*3.18*	*3*	*201.22*	*.31*	*.31*	*.36*
Does the survival (2 OM/1 AN 1 OM/4 OM) of eggs on a plant depend on the number of eggs on the plant, the treatment and the plant genotype (Loden, 78183)?										
M8	GLMM (Binomial)	Plant/Obs	Survival (survived/predated)	Intercept	*32.72*	*1*	*223.21*	*.28*	*.28*	<.001
				Genotype	*13.38*	*1*	*223.21*	*.28*	*.28*	<.001
				Eggs on plant	*1.78*	*1*	*223.37*	*.30*	*.30*	*.18*
				Treatment	*4.43*	*2*	*222.93*	*.37*	*.37*	*.10*
				Eggs on plant × Treatment	*0.12*	*2*	*226.80*	*.36*	*.36*	*.93*

The largest number of eggs were laid on the *S. viminalis* genotypes (part one: 78183, 78021; part two: 78183), and fewer eggs were laid when *A. nemorum* was present than when it was not (Table 1: M3; Figure 1c); the remaining omnivore treatments had similar effects on the number of eggs laid (Table 1: M4; Figure 1d).

**Figure 2:**
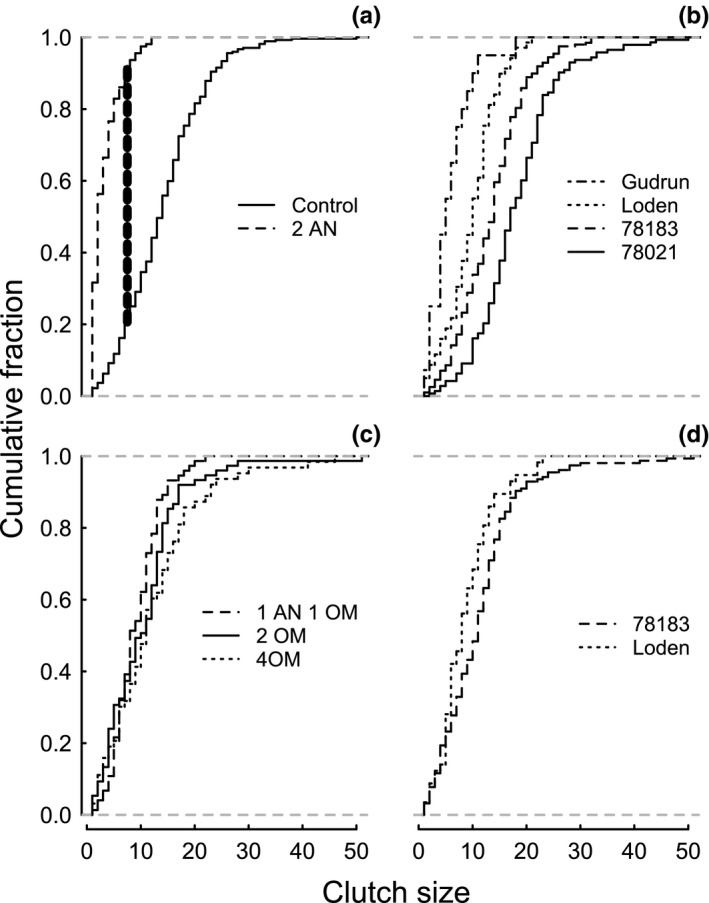
Cumulative fraction plot showing the relative distribution of clutch sizes from the first (a, b) and second (c, d) parts of the experiment (Treatments: Control = only leaf beetles, AN = *Anthocoris nemorum*, OM = *Orthotylus marginalis*; Plant genotypes: 78183, 78021, Loden, Gudrun). All treatments reveal (a, c) smaller clutch sizes with omnivores present compared to absent and decreasing egg clutch size from genotype 78021 to genotype Gudrun (b) for the same cumulative fraction. Clutch sizes tend to be larger on genotype 78183 than on Loden in the second part (d). The bold broken vertical line indicates exemplary the point of maximal separation D (KS test)

Taking a closer look at the actual distributions revealed that the presence of *A. nemorum* changed the frequency of clutch sizes, leading to less variable and smaller clutch sizes when the omnivore was present compared to when it was absent, for the same cumulative fraction (e.g., clutch size at fraction 0.5: Control: 17.5; 2 AN: 6.5). In fact, this was true for all omnivore treatments (Figure 2a, c; *p*‐values (Bonferroni–Holm corrected)/D‐values: Control vs. 2 AN: <0.001/0.71, Control vs. 2 OM: <0.001/0.38, Control vs. 1 AN 1 OM: <0.01/0.25, Control vs. 4 OM: <0.05/0.19). The largest difference was found for *A. nemorum*, where 92% of the clutches were smaller than the point of maximum separation, while only 21% were smaller when there was no omnivore. The clutch size distribution was also specific for each genotype (Figure 2b, d), with decreasing variability and size for the same cumulative fraction from genotype 78021 to Gudrun in the first part of the experiment (78021 vs. 78183: <0.001/0.28; 78021 vs. Loden: <0.001/0.55; 78021 vs. Gudrun: <0.001/0.77; 78183 vs. Loden: <0.001/0.32; Loden vs. Gudrun: <0.01/0.44), while in the second part, the distributions tended to differ between 78183 and Loden (=0.07/0.20).

**Figure 3:**
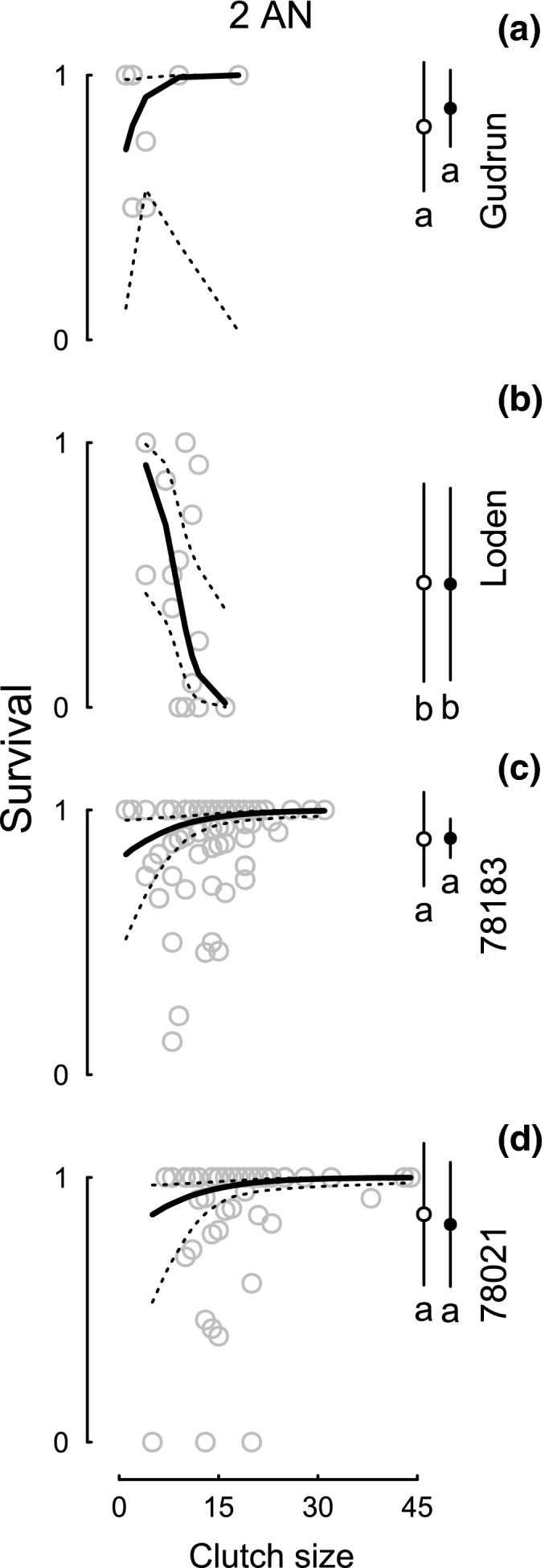
Individual egg survival within clutches in the presence of the omnivore *Anthocoris nemorum* in relation to clutch size for all four plant genotypes (*Salix dasyclados*: Gudrun, Loden; *Salix viminalis*: 78021, 78183). Gray circles show the proportion of surviving eggs within the clutch, and the lines indicate the model predictions with bootstrapped confidence limits. Black circles on the sides show the mean (± SD) survival per clutch (open) and per cumulative number of eggs on a plant (closed) for each plant genotype. Letters indicate differences between genotypes (*p* < .05; Tukey's test)

**Figure 4:**
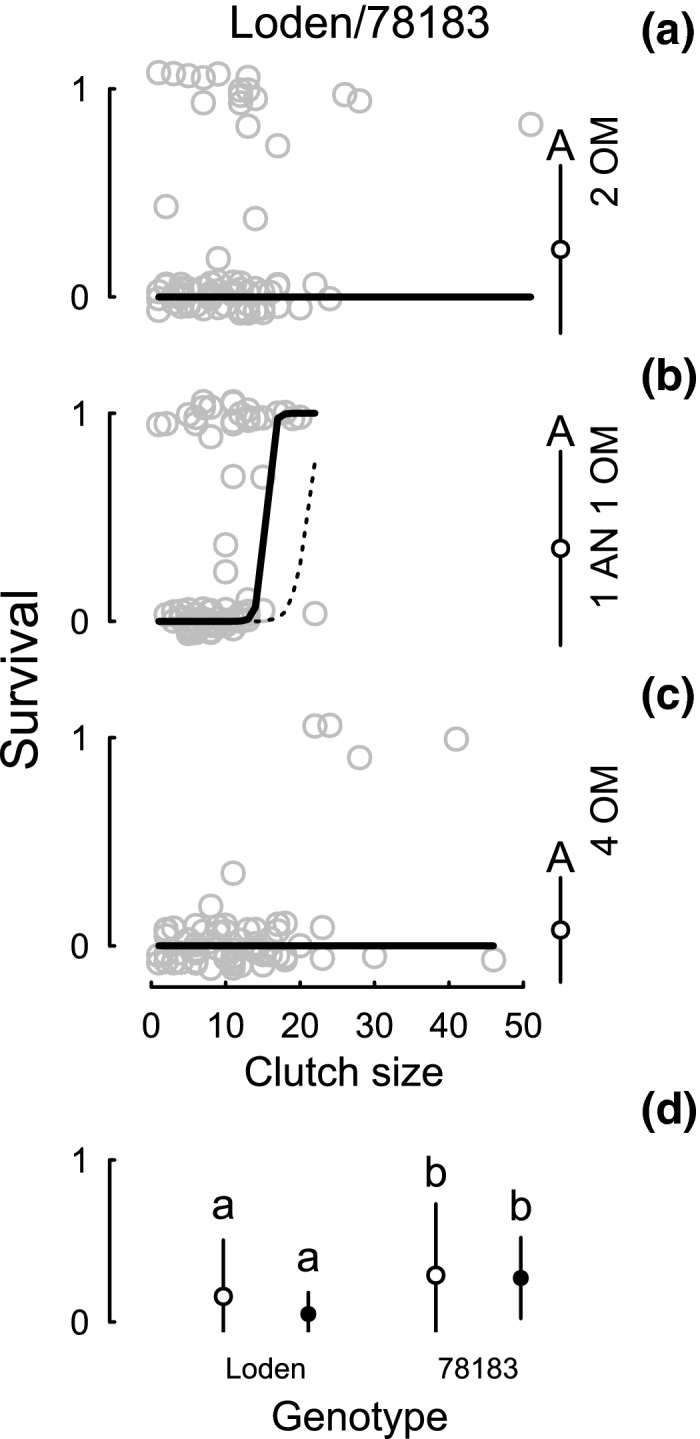
Individual egg survival within clutches (a‐c) in relation to clutch size for both plant genotypes (Loden, 78183) and omnivore treatments (AN = *A. nemorum*, OM = *O. marginalis*). Proportion of surviving beetle eggs is jittered to increase visibility, and the lines show the model prediction with bootstrapped confidence limits. The lowest figure (d) shows the overall mean of all three treatments. Black circles show the mean (± SD) survival per clutch (open) and per cumulative number of eggs on a plant (closed). Capital letters indicate differences between treatments, and lowercase letters differences between genotypes (*p* < .05; Tukey's test)

The egg survival in clutches (Table 1: M5) and the survival of all eggs on a plant (Table 1: 1 M7) in the presence of *A. nemorum* depended on the plant genotype, with lower survival on Loden (Figure 3). Egg survival was generally lower in the second part of the experiment and did not differ significantly between treatments (Figure 4a–c) although, again, it was higher for 78183 than for Loden (per clutch and per eggs on plant; Figure 4d). During the second part of the experiment, we found a tendency for genotype to have an effect on the egg survival in clutches and a significant interaction between clutch size and treatment (Table 1 1: M6). This interaction, and the interaction between clutch size and genotype in the first part (Table 1: M5), can be attributed to survival increasing with clutch size for the 1 AN 1 OM treatment (Figure 4) and for genotypes 78183 and 78021 (Figure 3), respectively. Egg survival was generally lower in the second part of the experiment which we attribute to the use of third instars of *O. marginalis*, as mentioned previously.

**Figure 5:**
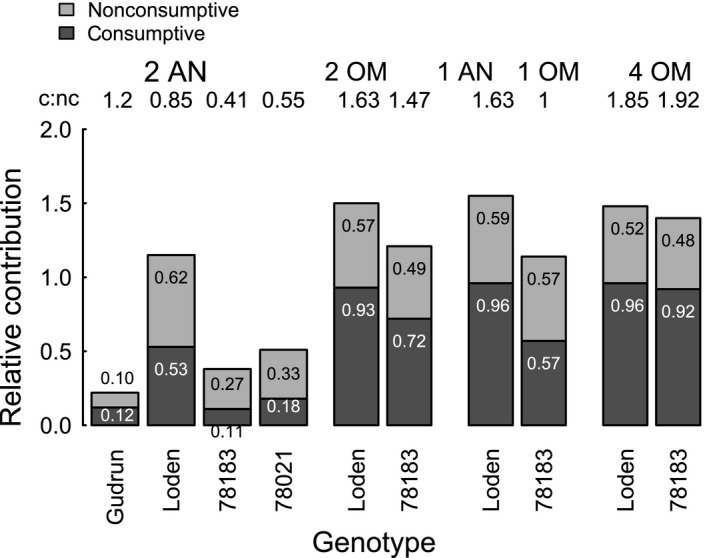
Contributions of consumptive and nonconsumptive effects on survival of herbivore eggs depending on omnivore treatment (AN = *Anthocoris nemorum*, OM = *Orthotylus marginalis*) and plant genotype (*S. dasyclados*: Gudrun, Loden; *S. viminalis*: 78021, 78183). The consumptive effect is expressed as the proportion of predated eggs of the total number of laid eggs on a plant and the nonconsumptive effect as the proportion of eggs not laid in the presence of omnivores compared to their absence (numbers within bars, respectively). The numbers above the bars express the consumptive: nonconsumptive ratio (for each genotype–treatment combination)

Visualizing the consumptive and nonconsumptive effects for all omnivore treatments revealed that the plant genotype affected not only the consumptive effect (egg survival), but also the nonconsumptive effect (eggs not laid due to omnivore presence) (Figure 5). The nonconsumptive effect ranged from 0.10 (= 10% fewer eggs laid in the presence of omnivores) to 0.62 and was generally larger on Loden than on genotype 78183. The c:nc ratio ranged from 0.33, indicating a nonconsumptive effect approximately three times greater than the consumptive effect, over exactly the same contributions (ratio of 1), to a consumptive effect about twice that of the nonconsumptive effect (1.92).

## 4 Discussion

As hypothesized, the mean clutch size of *P. vulgatissima* is driven by plant genotype, with larger egg clutches on the suitable (78183, 78021) than on the resistant genotypes (Gudrun, Loden). Based on studies of Lepidoptera, adjustment (Pilson & Rausher, 1988) or no adjustment (Janz & Thompson, 2002) could have been expected. Although this beetle matches its distances between clutches on a plant to leaf area (Stephan et al., 2015), here, as in other studies (Kagata & Ohgushi, 2002), we observed that clutch size depends on plant suitability (otherwise we would have seen larger clutches on Gudrun/Loden with larger leaves). Theoretical (Pilson & Rausher, 1988) and experimental evidence (Freese & Zwölfer, 1996) supports the interpretation that the adaptive mechanism is to reduce intraspecific exploitative competition (larvae feed gregariously close to their hatching site until the third instar) on suitable plant genotypes that support more larvae in an equivalent feeding area. Females therefore anticipate and match larvae number to food quality to reduce the disadvantages associated with aggregation. We also found changes in variability and frequency distribution of clutch sizes. Other than one previous study on a leaf mining moth and two host plant species showing frequency changes due to interspecific differences in leaf area (Kagata & Ohgushi, 2002), this is the first quantification of a frequency change due to plant suitability. Reducing intraspecific competition is therefore achieved by laying generally smaller clutches, and more of them. Such frequency changes may be important in overcoming plant defenses, which is probably not linearly related to group size.

Unexpectedly, the females did not change their mean clutch size due to omnivore presence. However, the increase in clutch size with number of eggs laid on a plant was stronger when *A. nemorum* was present than when not, indicating that the beetles do respond to the omnivores. This was confirmed by showing that either of the omnivore types/combinations reduced the median and the variation in clutch size and clutch size was always smaller for the same cumulative fraction. Thus, although females that perceived an omnivore had a lower oviposition rate associated with smaller clutch size, we observed that more smaller clutches were laid in the presence of omnivores (relative to the specific variation). This consistent response indicates that the beetles may not discriminate between the omnivores, which is against our expectations, especially as we confirmed that egg survival increases with clutch size for *A. nemorum* and also depends on plant genotype. However, for *O. marginalis,* egg survival was independent of clutch size (due to different strengths of attack‐abatement effects, see Stephan, Low, et al. (2016)) supporting our understanding of the functional differences between this omnivores. Changes in mean clutch size in the presence of predators have been reported for a moth (Subinprasert & Svensson, 1988). However, besides frequency changes exhibited by a butterfly that increases indirect predator protection by ants that guard mistletoes with aphids (Atsatt, 1981), this is the first attempt to look thoroughly at changes in frequency distribution of clutch sizes. Generally, looking at this measure and not only changes in mean is important as bottom‐up, lateral, and top‐down mechanisms may change if, for example, more smaller clutches are laid.

Laying fewer eggs on unsuitable plant genotypes and in the presence of omnivores is another behavioral response that may be more important for egg survival than clutch size modulation. We neither evaluated the oviposition choice in a field set up (Tschanz, Schmid, & Bacher, 2005) nor with alternative host plants, but employed a no‐choice assay. However, oviposition rate is a fitness proxy in this species and our results demonstrate that host acceptance is higher on suitable plant genotypes (Lehrman et al., 2012; Stenberg et al., 2010). Most interesting was that omnivore presence also reduces the beetles’ oviposition rate, making it the key behavioral response in this species, depending on aggregation level on the plant (Stephan et al., 2015), host plant suitability, and predators/omnivores. The lower oviposition rate in the presence of *A. nemorum* seemed to be repeated at a similar strength in the second part of the experiment for all three omnivore treatments. Predatory mite eggs can trigger lower oviposition rates in herbivorous insects (Walzer & Schausberger, 2009), and the presence of an intraguild predatory mite can cause egg retention by a phytoseiid mite (Montserrat et al., 2007). Predator presence lowers the population growth of aphids (Kersch‐Becker & Thaler, 2015; Nelson, 2007; Nelson et al., 2004), affects oviposition site choice by mosquitoes (Blaustein & Kotler, 1993), and even overrides inferior plant quality in beetles (Ballabeni, Wlodarczyk, & Rahier, 2001). However, our results seem to be the first report of predator presence lowering oviposition rate of individual herbivorous insects, which represents the most direct measure of a nonconsumptive effect on fitness.

In calculating the c:nc ratio, we found that the strength of the nonconsumptive effect ranged from one‐third up to twice as strong as the consumptive effect, depending on plant genotype and omnivore treatment. In other words, the presence of the omnivore reduced the oviposition rate by at least one‐third compared to its egg consumption. Looking at the contributions of this top‐down effect on the prey, we saw that the nonconsumptive effect was larger on Loden compared to 78183 (and 78021) for *A. nemorum*. The higher consumption is most likely due to lower plant quality perceived by the omnivore leading to diet and behavior adjustment (less plant food; more intense hunting and increased encounters with ovipositing females), which also results in the higher nonconsumptive effect. This behavioral change may depend on the prey density as consumptive and nonconsumptive effects were lowest on Gudrun, where switching to more intense hunting may not been triggered due to low egg clutch numbers and therefore very low numbers of encounters. The intensification of disturbance and a higher nonconsumptive effect due to low plant quality was even strong for *O. marginalis* (the higher consumptive effect was probably due to older and larger individuals). This greater disturbance may have been related to the greater overlay of oviposition preferences and preferred hunting area on the vertical shoot axis and is/will be addressed elsewhere (Stephan, 2016; Stephan J. G., Stenberg J. A., & Björkman C., unpublished).

**Figure 6:**
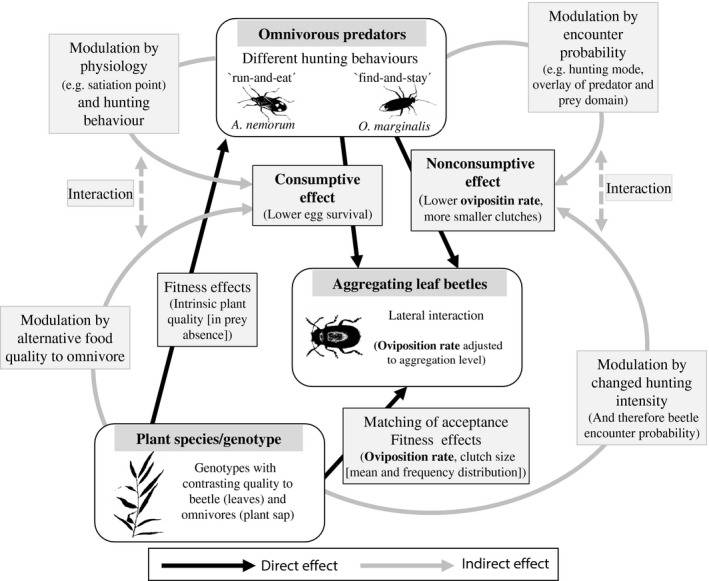
Overview of the direct and indirect effects that change the consumptive and nonconsumptive effect of omnivores on the reproductive behavior of the leaf beetles. Oviposition rate (here: a proxy for leaf beetle fitness) is affected by bottom‐up, lateral (Stephan, Low, et al., 2016), and top‐down factors

The relative contribution (a measure independent of prey density) of the nonconsumptive effect on the prey was, with the exception of the last treatment with four *O. marginalis*, consistently lower (higher c:nc ration) on Loden/Gudrun than 78183/78021. This means that increased egg consumption on plants of low quality to the omnivore (Loden/Gudrun) is accompanied by less time spent searching for prey and thus less disturbance of the ovipositing leaf beetle females. Consequently, although the plant genotype gains protection through egg consumption (besides lower herbivore acceptance), there might also be a “cost” in the form of lower benefits from the nonconsumptive effect. This indirect pathway associated with plant genotype extends the tritrophic interaction (Figure 6) and probably depends on the omnivore density and inter‐ and intraspecific interactions among the omnivores (here: neutral interference between the omnivores and between *O. marginalis*, and negative interference between *A. nemorum* (Björkman & Liman, 2005)).

Another important outcome of this study is to highlight the need to investigate whether a predator/omnivore could act as an indirect plant/genotype defense by including the herbivore and its (avoidance) behavior in future experiments. Therefore, not only intrinsic sap quality, but also foraging kairomones from beetles (Fernandez & Hilker, 2007) or plant volatile induction due to feeding or oviposition (Dicke & Baldwin, 2010) that may change the predator/omnivore behavior need to be considered.

We have yet to determine the real contributions of plant genotype‐mediated changes to nonconsumptive effects and the ultimate outcome to the plant. In many biological control strategies, pest density is estimated by counting the individuals or assessing the damage and relating it to the predator/parasitoid density/diversity under consideration of spillover and dilution effects (Andow, 1991; Stephan, Albertsson, Wang, & Porcel, 2016). However, after accounting for these, care should be taken to link any desired pest control to consumption directly, as there may be a nonconsumptive component. Although it is probably difficult to detect whether predation risk would be lower on specific plant genotypes (Stephan, Albertsson, et al., 2016), we did find some preliminary evidence. Certainly, the influence of retaining eggs or delaying oviposition still needs to be evaluated as many other factors, including habitat heterogeneity (Andersson, Löfstedt, & Hambäck, 2013), valuing an individual's own performance higher than that of its offspring (Mayhew, 2001), and higher predation risk on otherwise suitable hosts (Egusa, Nishida, Sawada, & Fujisaki, 2008), may override this positive effect on fitness. Anyhow, the concept of indirect defense and its application as a biocontrol would gain by considering nonconsumptive effects of different predators/omnivores on different host plants. Regarding the outcome to the plant, we found that the highest host acceptance by the beetle occurred on the genotypes with the lowest c:nc ratio (78183/78021). High damage due to intrinsic herbivore host acceptance is therefore reinforced by a low nonconsumptive effect from the omnivore providing plant protection.

Recently, Kersch‐Becker and Thaler (2015) investigated the interaction between consumptive, nonconsumptive effects and host plant resistance, using genetically modified plants and predators with impaired mandibles. Herbivore‐induced plant volatiles may have changed the predator's (lady beetle) foraging behavior. Olfactometric assays with *A. nemorum* showed that a *S. dasyclados* genotype similar to genotype Loden is more attractive than a *S. viminalis* genotype similar to 78183 and 78021, but only if the plants were attacked by the beetle. Otherwise, both genotypes had a similar attractiveness, which was only slightly higher than that of ambient air (Lehrman, Boddum, Stenberg, Orians, & Björkman, 2013). Clearly plant volatiles play a role in *A. nemorum*, and probably *O. marginalis* behavior, but how important they are in relation to alternative plant food is unclear. More importantly, with more natural host plants (commercial clones, but *Salix* naturally hybridize) and unharmed omnivores, we could show how key reproductive traits of *P. vulgatissima* change due to the quality of different hosts and the presence of predacious omnivores with different foraging behaviors. Considering that omnivores may be more common than strict carnivores (Rosenheim & Corbett, 2003), shape food webs (Holt & Polis, 1997), and ecosystem functions (Zhang, Richardson, & Negishi, 2004) and are important in biological control (Wäckers, 2005), this could be a pathway of fundamental importance to advance ecological theory and associated applications.

In summary, increasing clutch size in response to omnivores in cases where it would benefit egg survival does not occur in our system, and other behavioral adjustments may be more important. Because nonconsumptive effects may be at least a third as strong as the effects of consumption and because of the interaction between bottom‐up and top‐down effects, we wish to stress the importance of nonconsumptive effects in indirect plant defense and biocontrol. Our study illustrates the merits of using direct measures for fitness, including directly relating consumptive and nonconsumptive effects to the specific omnivore type/combination and the specific plant genotype. Because oviposition rate and clutch size are key life‐history traits for reproduction, understanding their modulation by bottom‐up and top‐down effects will help us to understand how and why species aggregate.

## Data Accessibility

The data will be uploaded at Dryad.

## Conflict of Interest

None declared.
