# Supplementary material for: Consumptive and nonconsumptive effect ratios depend on interaction between plant quality and hunting behavior of omnivorous predators
Source: Ecol Evol. 2017 Mar 9;7(7):2327–39. doi: 10.1002/ece3.2828 (PMC5383501; doi:10.1002/ece3.2828)
Supplement: Supplementary file 2 [file ECE3-7-2327-s002.docx]

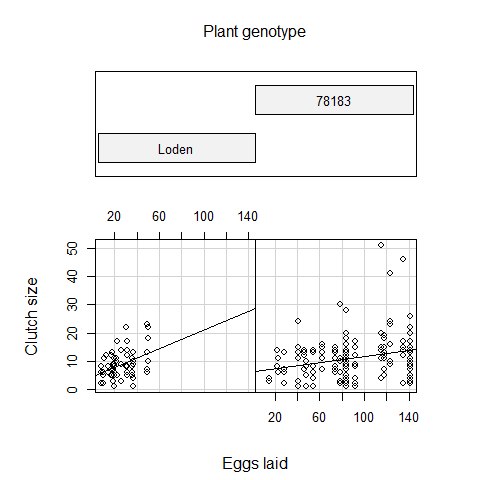


Fig. S2: Clutch size in relation to number of eggs laid on a plant from the second part of the experiment (Control = only leaf beetles, AN = *Anthocoris nemorum*, OM = *Orthotylus marginalis*). On the Loden genotype, the clutch size increases more steeply with number of eggs laid on a plant. Lines indicate linear predictions.
